# Children in hospital in Ireland - what do they eat and what do they weigh: a cross-sectional study

**DOI:** 10.1186/1756-0500-5-491

**Published:** 2012-09-06

**Authors:** Aisling Flinn, Alan P Macken, Walter Cullen, Des Leddin, Colum Dunne, Clodagh S O’Gorman

**Affiliations:** 1 The Children’s Ark, University Hospital, Limerick, Ireland; 2 National Children’s Research Centre, Dublin, Ireland; 3 Centre for Interventions in Infection, Inflammation & Immunity (4i), Graduate Entry Medical School, University of Limerick, Limerick, Ireland; 4 Dalhousie University, Halifax, Canada; 5 Graduate Entry Medical School/Scoil Leighis Iontrála Iarchéime, Faculty of Education & Health Sciences/Dámh an Oideachais agus na nEolaíochtaí Sláinte, University of Limerick/Ollscoil Luimnigh, Limerick/Luimnigh, Ireland

**Keywords:** Overweight, Obesity, Children, Hospital, Nutrition

## Abstract

**Background:**

Overweight and obesity is a growing problem in Ireland. Many parents are unaware when their child is overweight or obese. Our objectives were to examine parents’ perceptions of a healthy diet and their children’s BMI; and to evaluate the food offered to children in our paediatric in-patient unit.

**Findings:**

A retrospective questionnaire was distributed to 95 patients and their families admitted over one month. Seventy-eight had BMI values calculated (42 males, 36 females). Twenty-one children (26.9%) were overweight/obese: 14/21 parents (66.7%) thought their child had a normal weight. Sixty percent of children served dinner in the hospital were given fried potatoes. Four had fruit/vegetables. Forty-six parents brought food into hospital, of these 14 brought purchased food.

**Conclusions:**

This study highlights the problem of child obesity in Ireland and parental underestimation of this problem. The nutritional value of food served to children in hospital needs to be improved and hospital admissions used as opportunities to promote healthy eating habits.

## Findings

### Background

**Figure 1:**
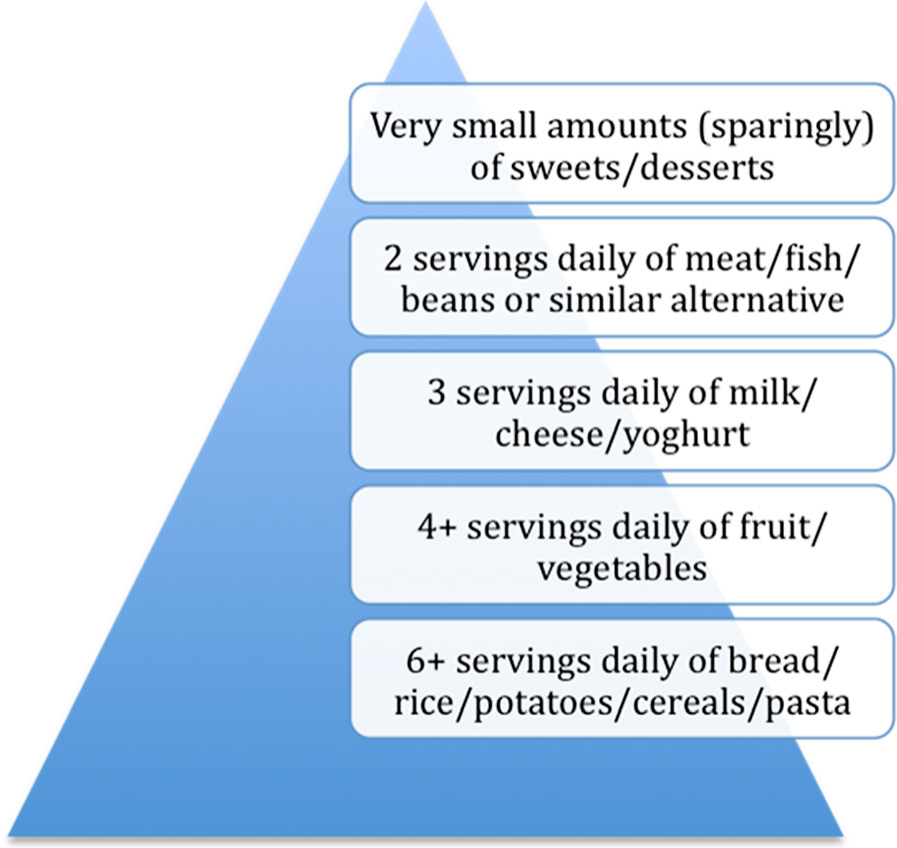
Irish DOHC Daily Diet Recommendations

Overweight and obesity in childhood are emerging as major public health challenges in Ireland. In 2002, 23% of boys and 28% of girls aged 4 - 16 years in the Republic of Ireland were either overweight or obese [1]. In 2011, the ‘Growing Up in Ireland’ study, a national longitudinal study of a cohort of 11,100 children, found that 19% and 6% of three-year-olds were overweight or obese, respectively [2]. Recognising the obesity “epidemic”, the National Taskforce on Obesity was launched by the Irish government in 2005 with an objective to ‘enable people through health promotion, prevention and care to achieve and maintain healthy eating and active living throughout their lifespan’ [3]. Closely aligned to the National Taskforce are a cluster of national nutritional guidelines including “Food and Nutrition Guidelines” for Pre-School Services (2004) [4] and Primary School Services (2003) [5], and “Guidelines for Developing a Healthy Eating Policy in Post-Primary Schools” (2008) [6] issued by the Irish Department of Health and Children (DOHC) as part of the Irish Government’s National Health Promotion Strategy (2000/2005). The aim was to promote nutrition awareness as well as healthy eating habits and a healthy body image among children (Figure 1). 

Current Irish DOHC recommendations also include limiting the intake of fried foods to 1 - 2 times per week and drinking at least 8 cups daily (one cup being equivalent to 8 ounces). In 2006, the American Heart Association recommended that children over 2 years should consume either low-fat or fat-free dairy foods [7]. The Irish DOHC and EUNUTNET (European Network for Public Health Nutrition: Networking, Monitoring, Intervention Training) recommend that low-fat milk is not suitable for children under 2 years because of the lower energy and fat-soluble vitamin content [8]. The Irish DOHC states that low-fat milk can be introduced after 2 years for children with a good varied diet, but does not recommend fat-free milk for children under 5 years.

Prevention and treatment of obesity in children requires parental involvement and support [9,10]. Such involvement, in turn, is multifaceted and includes parental ability to recognise that their child is overweight or obese, an understanding of, and vigilance for, associated health risks, understanding of the principles of nutrition and the ambition to provide healthy nutritionally balanced meals. Barriers to success include lack of parental involvement and motivation and lack of professional support services [11]. However, many parents underestimate their child’s weight [12,13]. In a study based in New York involving questionnaires distributed at children’s well-care visits, 19/83 (23%) parents surveyed had overweight children but 17/19 parents (89.5%) underestimated their child’s weight [14]. A meta-analysis of several studies from the United Kingdom, Australia, Italy and the United States also highlighted parental underestimation of their children’s weight and found that overweight parents were most likely to underestimate their children’s weight [15].

There are approximately 144,700 paediatric hospital admissions annually in Ireland. Children in hospital should be a “captive” and receptive audience for opportunistic reinforcement of many aspects of healthy living, including the making of healthy food and lifestyle choices. However, one Canadian study demonstrated that inpatient children’s intake of fruit and vegetables was below the recommended intake range according to the Canada Food Guide [16] and that there was also excessive intake of energy-dense foods with low nutritional value [17]. In Ireland, 'Food and Nutritional Care in Hospitals - Guidelines for Preventing Under-Nutrition in Acute Hospitals' were published by the Irish DOHC in April 2009 and focused on adult patients but state that children in acute hospitals 'may benefit from the introduction of guidelines' [18]. The aims of our study were: (i) to examine parents’ perceptions of a healthy diet and their children’s BMI; and (ii) to evaluate the food offered to children admitted to a paediatric ward based in a regional centre.

### Methods

A questionnaire was developed, validated and then administered by a single investigator to 95 consecutive medical and surgical paediatric patients and their families, who were admitted to The Children’s Ark Mid-Western Regional Hospital (MWRH) Limerick from 16th August to 10th September 2010. The Children’s Ark is a 53-bed general paediatric unit with two wards and has 3,500 admissions annually. The children are served breakfast, lunch, dinner and a bedtime snack daily, with two options available for each meal. The questionnaire was completed based on parental recall.

Information collected from the questionnaires included: demographic details, food types and quantity eaten in the hospital and at home, parent’s estimation of their child’s height and weight, and whether they thought their child was overweight/underweight/normal weight, parents’ knowledge of ideal daily intake of various food products and fat content of milk, and what food the parents purchased or brought into the hospital for their child to eat, and why (Additional file 1).

Exclusion criteria included (1) age less than 2 years; (2) age over 14 years; and (3) patients receiving nutritional supplements eg. nasogastric feeds, PEG feeds or total parenteral nutrition. Patients aged 2 to 14 years admitted overnight or longer were eligible for inclusion. Children with chronic medical conditions such as cystic fibrosis and children with no previous medical history were both included. Children were measured on admission and the BMI value was calculated (kg/m^2^). BMI values were classified as underweight, healthy weight, overweight or obese according to the Centre for Disease Control and Prevention BMI centile guidelines [19]. Ethical approval (from the Mid-Western Regional Hospital complex ethics committee) was obtained prior to starting the study.

### Results

Ninety-five questionnaires were completed for 53 male and 42 female patients. Seventy-five were completed by the child’s mother, 16 by the child’s father and 4 by another family member. The mean age of all the children was 7.0 years (±3.7). The most common reason for admission was an orthopaedic problem (N = 14). Other reasons for admission included a respiratory problem (N = 12), appendicitis/abdominal pain (N = 10), vomiting/diarrhoea (N = 7), and viral illness (N = 6) (see Table 1). In-patients with chronic diseases included 3 with type 1 diabetes mellitus, 2 with cystic fibrosis, 2 with malignancies, 1 patient with chronic pain and 1 with spinal muscular atrophy. All had previous hospital admissions also.

**Table 1:** Reasons for admission to hospital

**No Patients**	**Reason for admission**
14	Orthopaedic problem
7	Vomiting/diarrhoea
6	Viral illness
5	UTI
5	Pneumonia/respiratory problem other that asthma/wheeze/CF
5	Elective surgical admission (T’s and A’s mostly)
5	Asthma exacerbation/wheezy episode
5	Appendicitis/suspected appendicitis
5	Abdominal pain
4	Head injury
3	Tonsillitis
3	Seizures
3	Diabetes
3	Cellulitis/abscess/skin infection
2	Suspected testicular torsion
2	Rash
2	Musculoskeletal problem
2	Malignancy
2	Injury to abdomen
2	Ingestion/inhalation of a foreign body
2	CF exacerbation
1	Thyroid cyst
1	Post-operative complication
1	Polyuria
1	Management of chronic pain
1	Malaria
1	Laceration
1	Constipation
1	Allergic reaction

### Estimation of overweight and obesity

Seventeen patients did not have both a height and weight measured, therefore a BMI value could not be calculated. BMI values were calculated for 78 patients (42 males and 36 females) and 21/78 children (26.9%) were either overweight or obese. When the patients with chronic medical conditions were excluded, the number of obese or overweight children was 29.2% (21/72).

**Figure 2:**
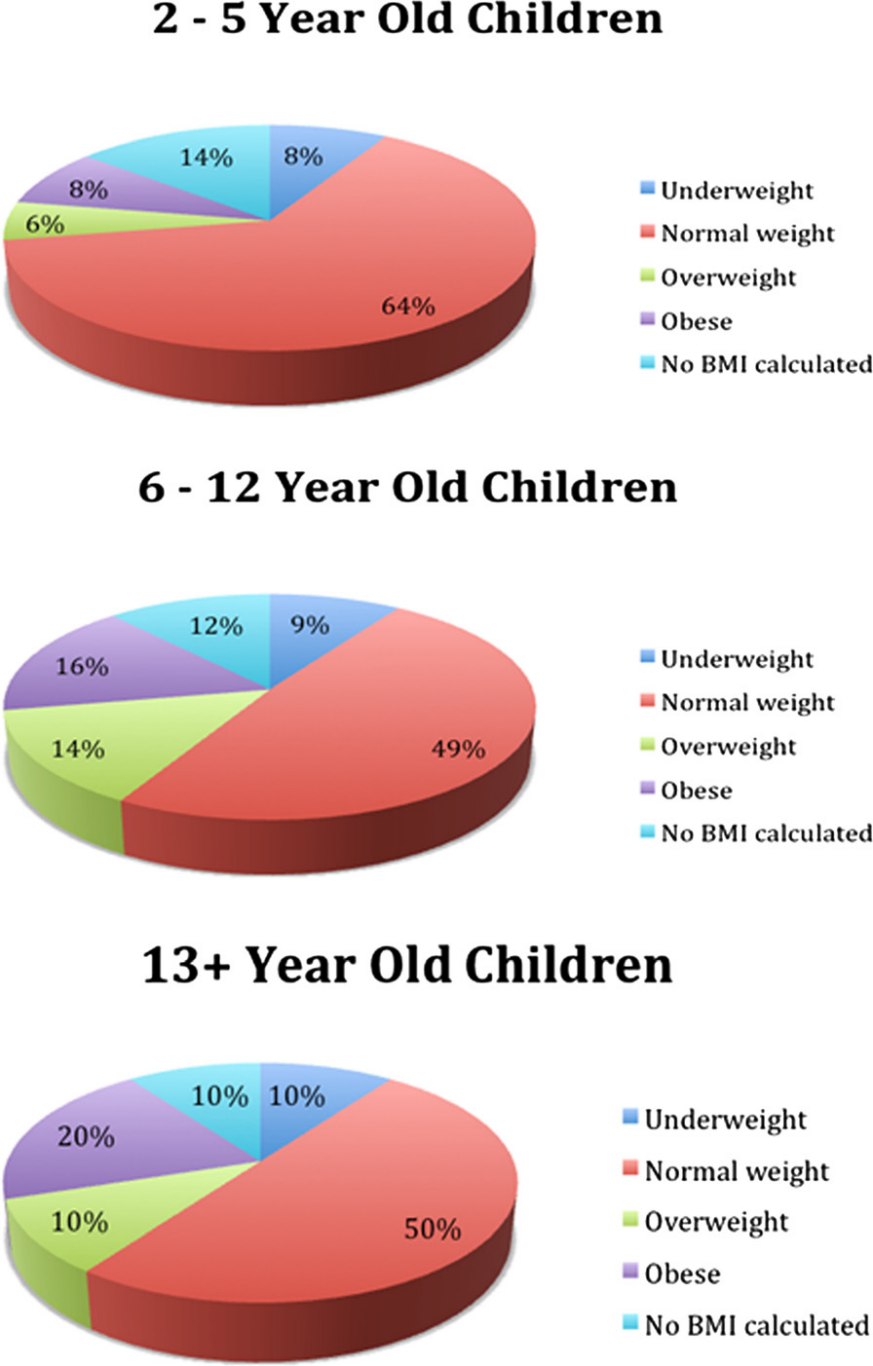
Estimation of overweight and obesity according to age groups (of the 95 children in total)

Of the 42 males, 24 (57.1%) were of a healthy weight and 10 (23.8%) were overweight or obese. Of the 36 females, 25 (69.4%) were of a healthy weight and 11 (30.6%) were overweight or obese. Looking at the different age groups, 16.1% of 2 - 5 year old children, 34.2% of 6 - 12 year old children, and 33.3% of children 13 years or older were overweight or obese (See Figure 2 and Table 2).

**Table 2:** Overall breakdown of underweight/normal weight/overweight and obese children

**Gender**	**Age group**	**Total**	**Underweight**	**Normal weight**	**Overweight**	**Obese**
Female	All ages	36	0	25 (69.4%)	5 (13.9%)	6 (16.7%)
	2 - 5 years	12	0	8 (66.7%)	2 (16.7%)	2 (16.7%)
	6 - 12 years	20	0	14 (70%)	2 (10%)	4 (20%)
	13+ years	4	0	3 (75%)	1 (25%)	0
Male	All ages	42	8 (19%)	24 (57.1%)	4 (9.5%)	6 (14.3%)
	2 - 5 years	19	3 (15.8%)	15 (78.9%)	0	1 (5.3%)
	6 - 12 years	18	4 (22.2%)	7 (38.9%)	4 (22.2%)	3 (16.7%)
	13+ years	5	1 (20%)	2 (40%)	0	2 (40%)
Total	2 - 5 years	31	3 (9.7%)	23 (74.2%)	2 (6.4%)	3 (9.7%)
	6 - 12 years	38	4 (10.5%)	21 (55.3%)	6 (15.8%)	7 (18.4%)
	13+ years	9	1 (11.1%)	5 (55.6%)	1 (11.1%)	2 (22.2%)

### Parental perception of overweight and obesity

Of the 21 children who were either overweight or obese, 14 parents (66.7%) thought their child had a normal weight, one parent was unsure and only 6 parents (28.8%) recognized that their child was overweight.

### Evaluation of food offered to hospital inpatients

Fifty-six parents (58.9%) considered the food served in hospital to be similar to meals served at home. Of the 39/95 parents who said that the hospital food was different, the most common reason was that they would eat more fruit, salad or vegetables at home. Other examples of differences given by parents included: more variation at home (N = 8), would eat less chips at home (N = 5), and smaller meals at home (N = 2).

A total of 68/95 children ate breakfast served by the hospital. Cereal (73.5%) and/or white toast or bread (67.7%) were most commonly eaten. Several parents noted that no brown bread was offered. Sixty parents reported that their child had eaten lunch served by the hospital. Forty-nine children (81.7%) had meat (chicken/beef/bacon/ham/sausages/fish) and 34 (56.7%) had potatoes. Only 31.7% of children had vegetables and 3.3% had fruit. Thirty-five children had eaten dinner served by the hospital and 60% of these children had been given chips or potato wedges. Only 2/35 children (5.7%) had vegetables and, similarly, only 2/35 (5.7%) ate fruit.

Of the 68 children who ate breakfast, only 4/68 (5.9%) consumed yoghurt. However, 50/68 (73.5%) ate cereal, most likely with full-fat milk although this was not specifically asked. For lunch, 2 children (3.3%) had a serving of dairy and again at dinner only 3 children (8.6%) had a serving of dairy. Several parents also noted that no water was offered to the children with their meals. The only drinks noted to be served with meals were juice, milk or tea.

Forty-eight parents acknowledged that they brought food into the hospital for their child. Fourteen (29.1%) parents had brought food from a nearby take-away. The most common reason given by those as to why they brought in food for their child was because, or in case, their child did not like the hospital food (N = 17). Thirty-three parents had brought food into the hospital for snacks. The most popular snack brought in by parents was fruit (N = 14) followed by sweets, biscuits, chocolate, popcorn or crisps (N = 10). Eleven (22.9%) of the 48 children were either overweight or obese. Among the 47 children whose parents did not bring food into the hospital for them to eat, 10 (21.3%) were either overweight or obese.

When asked if they would encourage their child to eat hospital food if offered a healthy option, 84 parents reported that they would (Table 3).

**Table 3:** Daily Diet Recommendations

	**DOHC Guidelines**	**Options from questionnaire**	**Parents’ response (Total = 95)**
Fruit and vegetables	4+ servings of fruit/fruit juice/vegetables daily	0-1 servings/day	5 (5.3%)
		1-2 servings/day	7 (7.4%)
		2-3 servings/day	14 (14.8%)
		3-4 servings/day	18 (18.9%)
		4+ servings/day	49 (51.5%)
		No answer	2 (2.1%)
Fried foods	Limit intake to 1-2 times per week	0-1 servings/day	80 (84.2%)
		1-2 servings/day	8 (8.4%)
		> 2 servings/day	3 (3.2%)
		No answer	4 (4.2%)
Sweets and desserts	Should be eaten in small amounts (sparingly)	0-1 servings/day	67 (70.5%)
		1-2 servings/day	19 (20%)
		2-3 servings/day	5 (5.3%)
		3-4 servings/day	3 (3.2%)
		No answer	1 (1%)
Fish/poultry	2 servings daily of meat/fish/beans or alternative	0-1 servings/day	44 (46.3%)
		1-2 servings/day	41 (43.2%)
		2-3 servings/day	6 (6.3%)
		3-4 servings/day	1 (1%)
		4-5 servings/day	1 (1%)
		No answer	2 (2.1%)
Red meat	2 servings daily of meat/fish/beans or alternative	0-1 servings/day	60 (63.1%)
		1-2 servings/day	26 (27.4%)
		2-3 servings/day	6 (6.3%)
		3-4 servings/day	3 (3.2%)

Eighty percent of parents thought that their child should drink regular full-fat milk rather than low-fat milk, 14.7% thought that their child should drink low-fat milk and 5.3% of parents did not know.

## Conclusions

This study highlights the problem of child obesity in Ireland and the fact that parents underestimate this problem with only 28.8% of the parents questioned here recognising that their child was overweight or obese. Our study also brings attention to the types of food eaten by children in the hospital setting, in particular the abundance of chips and the lack of dairy products, fruit and vegetables. Our study has limitations. It is a questionnaire administered prospectively but based on parental recall, which therefore is likely to introduce recall bias. Furthermore, parents may have modified their responses due to their awareness that a paediatrician was administering the questionnaire. However, this would imply that their answers are more likely to overestimate the nutritional content of their children’s diets. It is also a small study; however it is a good representation of a population of paediatric in-patients in an Irish regional hospital. There was also incomplete recording of height and weight among patients thus limiting the numbers with BMI values included in our evaluation. Thirteen patients did not have a height, two patients did not have a weight and two patients did not have either a height or a weight measured. Six patients were unable to weight bear, one child refused to be measured and one oncology patient was in isolation. However, nine patients were discharged before a height and/or a weight was taken. This issue needs to be addressed by hospital staff as, not only is it a vital part of hospital admission which should be routinely documented, but it would also serve to reinforce to parents the importance of monitoring children’s growth. Although the reason for admission was documented, the length of admission was not recorded.

Four respondents were not parents. One was a significantly older sister, one was a grandmother and two were aunts all of whom were caring for the child while they were an inpatient and would also have cared for the child at some point at home as well, for example while the mother was at work. Before administering the questionnaire, each person was asked if, as the child’s caregiver at that time, were they confident about answering the questions and all responded that they were.

Children with chronic conditions were included in order to get a broader perspective of parent's opinions with regard to hospital food, from a representative general paediatrics population. We wanted to include those who would have had several admissions previously or a more prolonged admission and thus would have more exposure to the hospital food, while also including parents of otherwise healthy children for whom it would have been their first encounter with hospital food. It is likely that most of the children with chronic medical conditions would have received dietetic input previously, and so it is likely that these families had more knowledge about principles of nutrition in paediatrics. If these patients were excluded, the data would likely show poorer knowledge of nutrition. However, including these patients means that the data are representative of general paediatric inpatients and are generalisable to other general paediatric hospital populations.

Several parents commented that they would have been happy for their child to have chips and chicken nuggets for one night in hospital, but when they were admitted for a more prolonged period this choice of food and lack of variety and healthy options can become very frustrating.

One of the strengths of our study is that it is novel, with no similar study being done previously in Ireland. Additionally, it reflects parents’ opinions with regard to daily diet recommendations, the hospital food and their children’s weights. Our study may help encourage hospital staff to tackle the problem of child obesity while children are in hospital. Hospitals sometimes fail to encourage healthy eating to children and families and previous studies have shown that children make poor food choices even when given healthy food options [16]. Therefore, we suggest that Irish hospitals should improve the nutritional value of the food that is served to children in hospital and use hospital admissions as an opportunity to promote healthy eating habits. This study also suggests that parents need more education on healthy eating and the nutritional needs of their children. Routine recording of children’s height and weight by hospital staff, with calculation and explanation to parents of their child’s BMI centile, would provide an opportunity for health care professionals to communicate and reinforce healthy living principles to children and their parents.

## Competing interests

The authors declare that they have no competing interests.

## Authors’ contributions

AF was responsible for administering the questionnaire to patients and families, collecting, analyzing and interpreting the data and writing the manuscript. APM assisted in the analysis and interpretation of the data, and with critical revision of the manuscript. WC critically revised the manuscript and gave approval prior to submission. DL critically revised the manuscript and gave approval prior to submission. CD was responsible for the idea of the study, participated with design of the study and assisted in critical revision of the manuscript and gave approval prior to submission. CSO’G was also responsible for the idea of the study, the study design, developed the questionnaire, assisted in co-ordination and critical revision of the manuscript and gave approval prior to submission. All authors read and approved the final manuscript.

## Supplementary Material

Additional file 1 What Food Do Children in Hospitals Eat? Click here for file
