# Supplementary material for: Children in hospital in Ireland - what do they eat and what do they weigh: a cross-sectional study
Source: BMC Res Notes. 2012 Sep 6;5:491. doi: 10.1186/1756-0500-5-491 (PMC3441275; doi:10.1186/1756-0500-5-491)
Supplement: Additional file 1 — What Food Do Children in Hospitals Eat? [file 1756-0500-5-491-S1.doc]

**What Food Do Children in Hospitals Eat?**

**Study Investigators:**

**Dr Aisling Flinn, Paediatrics SHO**

**Dr Alan Macken, Paediatric Specialist Registrar**

**Prof Colum Dunne, Director of Research, UL Graduate Entry Medical School**

**Prof Clodagh O’Gorman, Consultant Paediatrician**

**Background information**

Date:

Questionnaire No:

Name of child admitted (optional): ___________________________________________

**Q1. Age of child:** (please fill in your child’s age in years in the box below)

[ ] years

**Q2. Gender of child:** (please tick)

Male [ ] Female [ ]

**Q3. Date of admission:**

| Day | Month | Year |
| --- | --- | --- |

**Q4. Who completed this form:** (please tick one box)

Mother [ ]

Father [ ]

Other [ ] please specify ­­___________________________________________

**Q5. Current diagnosis / reason for admission:**

­­

**Q6. Treatment required to date:**

Steroid therapy Yes [ ] No [ ]

**Q7. Are the types of foods your child has been eating while in hospital similar to the foods that they eat at home?** (Please tick one box)

Yes [ ]

No [ ]

Unsure [ ]

If no, please explain what is different?

**Q8. Is the amount of food that your child has been eating while in hospital similar to the amount food that they eat at home?** (Please tick one box)

Yes [ ]

No [ ]

Unsure [ ]

If no, please explain what is different?

**Q9. Is your child’s appetite similar in hospital to his/her appetite at home?** (Please tick one box)

Yes [ ]

No [ ]

Unsure [ ]

If no, please explain what is different?

**Q10. Do you know / can you estimate your child’s weight?** (Please fill in your child’s weight in the appropriate box)

[ ] kg *or* [ ] stones and [ ] lbs

**Q11. Do you know / can you estimate your child’s height?** (Please fill in your child’s height in the appropriate box)

[ ] cm *or* [ ] feet and [ ] inches

**Q12. Do you think that your child is:** (please tick one box)

Underweight for age [ ]

Normal weight for age [ ]

Overweight for age [ ]

Unsure [ ]

**Q13. What would be the average / an acceptable weight for a child of your child’s age and gender?** (Please fill in the weight in the appropriate box(es) below)

[ ] kg *or* [ ] stones and [ ] lbs

**Q14. In your opinion, should children of your child’s age drink low fat or regular fat milk?** (Please tick one box)

Low fat [ ]

Regular fat milk [ ]

No opinion [ ]

**Q15. In your opinion, how often should children of your child’s age eat/drink the following foods:** (please tick one box in each row)

|  | **Number of times per day** | | | | | |
| --- | --- | --- | --- | --- | --- | --- |
| **Type of Food** | **0 - 1** | **1 - 2** | **2 - 3** | **3 - 4** | **4 - 5** | **5+** |
| Red Meat | [ ] | [ ] | [ ] | [ ] | [ ] | [ ] |
| Fish or poultry | [ ] | [ ] | [ ] | [ ] | [ ] | [ ] |
| Fruit and vegetables | [ ] | [ ] | [ ] | [ ] | [ ] | [ ] |
| Fried Foods | [ ] | [ ] | [ ] | [ ] | [ ] | [ ] |
| Sweets/deserts | [ ] | [ ] | [ ] | [ ] | [ ] | [ ] |

**What is your child eating on the ward?**

**Q16. What food served on the ward by the hospital is your child eating?** (Please give short details of the following meals/snacks)

Breakfast ___________________________________________

Lunch ___________________________________________

Tea ___________________________________________

Bedtime snack ___________________________________________

Other snacks ___________________________________________

How much of the food did your child eat?

None [ ]

Small amount [ ]

Half [ ]

All [ ]

Were you offered choices?

Yes [ ]

No [ ]

If so, what made you choose the above foods?

Were you offered a healthy option?

Yes [ ]

No [ ]

Would you encourage your child to eat hospital food if they were offered a healthy option?

Yes [ ]

No [ ]

**Q17. What food brought into hospital is your child eating?** (Please give short details for the following meals/snacks)

Breakfast ___________________________________________

Lunch ___________________________________________

Tea ___________________________________________

Bedtime snack ___________________________________________

Other snacks ___________________________________________

Where did this food come from? ___________________________________________

Was it prepared at home? ___________________________________________

If so, by whom? ___________________________________________

If not, then where was it purchased? ___________________________________________

Why did you choose to bring food in to your child?

**Q18. What food purchased in the hospital is your child eating?** (Please give short details for the following meals/snacks)

Breakfast ___________________________________________

Lunch ___________________________________________

Tea ___________________________________________

Bedtime snack ___________________________________________

Other snacks ___________________________________________

Where did this food come from? ___________________________________________

Where was it purchased? ___________________________________________

Why did you choose to buy this food for your child?

**Q19.** **Have you any suggestions / comments regarding what food you would like to see**

**served to children on the ward?**

**Any other additional information you would like to offer?**

**Thank you for completing this questionnaire. We appreciate your time and effort.**

**Prof Clodagh O’Gorman**

**On behalf of all study investigators 30^th^ May 2010**
